# Increased B-type natriuretic peptide and decreased proteinuria might reflect decreased capillary leakage and is associated with a better outcome in patients with severe burns

**DOI:** 10.1186/cc10297

**Published:** 2011-07-01

**Authors:** Karina de Leeuw, Marianne K Nieuwenhuis, Anuschka S Niemeijer, Hans Eshuis, Gerard IJM Beerthuizen, Wilbert M Janssen

**Affiliations:** 1 Department of Internal Medicine, Martini Hospital, van Swietenplein 1, 9700 RM Groningen, The Netherlands; 2 Association of Dutch Burn Centres, Martini Hospital Groningen, van Swietenplein 1, 9700 RM Groningen, The Netherlands; 3 Burn Centre, Martini Hospital, van Swietenplein 1, 9700 RM Groningen, The Netherlands

## Abstract

**Introduction:**

It is difficult to adjust fluid balance adequately in patients with severe burns due to various physical changes. B-type natriuretic peptide (BNP) is emerging as a potential marker of hydration state. Proteinuria is used as a predictor of outcome in severe illness and might correlate to systemic capillary leakage. This study investigates whether combining BNP and proteinuria can be used as a guide for individualized resuscitation and as a predictor of outcome in patients with severe burns.

**Methods:**

From 2006 to 2009, 38 consecutive patients (age 47 ± 15 years, 74% male) with severe burns were included and followed for 20 days. All had normal kidney function at admission. BNP and proteinuria were routinely measured. Ordered and actually administered fluid resuscitation volumes were recorded. The Sequential Organ Failure Assessment (SOFA) score was used as the measure of outcome.

**Results:**

BNP increased during follow-up, reaching a plateau level at Day 3. Based on median BNP levels at Day 3, patients were divided into those with low BNP and those with high BNP levels. Both groups had comparable initial SOFA scores. Patients with high BNP received less fluid from Days 3 to 10. Furthermore, patients with a high BNP at Day 3 had less morbidity, reflected by lower SOFA scores on the following days. To minimize effects of biological variability, proteinuria on Days 1 and 2 was averaged. By dividing the patients based on median BNP at Day 3 and median proteinuria, patients with high BNP and low proteinuria had significantly lower SOFA scores during the entire follow-up period compared to those patients with low BNP and high proteinuria.

**Conclusions:**

Patients with higher BNP levels received less fluid. This might be explained by a lower capillary leakage in these patients, resulting in more intravascular fluid and consequently an increase in BNP. In combination with low proteinuria, possibly reflecting minimal systemic capillary leakage, a high BNP level was associated with a better outcome. BNP and proteinuria have prognostic potential in severely burned patients and may be used to adjust individual resuscitation.

## Introduction

An important feature of burn trauma is a massive loss of plasma from the intravascular to the extravascular space due to systemic microvascular leakage, which is triggered by inflammatory mediators [1]. Capillary leakage is massive during the first 12 to 16 hours, and then decreases. Because of this capillary leakage and vasodilatation in combination with alterations in cardiac function, it is difficult to maintain, monitor and adjust fluid balance in patients with severe burns [2].

Fluid resuscitation is vital in severely burned patients. Yet, resuscitation with too large volumes of fluid has several negative consequences, including compartment syndromes, conversion of superficial burns into deep burns and worsening of burn edema. Even potentially fatal complications can occur, such as pulmonary edema and intra-abdominal hypertension. Current markers of adequacy of resuscitation are normalization of serum lactate, urine production or invasive measurements [3]. However, clearance of serum lactate depends on adequate liver function, adequate renal function, and normal electrolyte levels. Furthermore, Papp *et al. *demonstrated that serum lactate as well as urine production could be normal despite the presence of hypovolaemia as measured by central venous pressure and pulmonary artery wedge pressure [4]. These last measurements might be the gold standard to determine hemodynamics; however, these measurements are invasive and not performed regularly in every hospital. Because of these limitations of the current markers to monitor resuscitation, other biomarkers, specifically markers that can be measured at the bedside, are needed.

An interesting marker might be serum B-type natriuretic peptide (BNP). BNP is secreted from myocardium under increased wall stretch and is used as a non-invasive method to detect heart failure [5-8]. Recently, Friese *et al. *showed an increase of BNP levels after resuscitation in trauma patients, suggesting it might be a marker of volume resuscitation after injury [3]. Increase of BNP levels during the first 72 hours of resuscitation has also been demonstrated in nine burn patients [4]. It can be hypothesized that an absence in increase of BNP levels, reflecting no increase in ventricular pressure, in severely burned patients despite high amounts of fluids indicates an inadequate resuscitation. The explanation of no increase in ventricular pressure might be the ongoing increased capillary leakage, causing a persistent low intravascular volume.

It would even be better to find a marker which reflects the massive capillary leakage present in patients with burns. Capillary leakage is one of the events in endothelial dysfunction [9]. Proteinuria, especially microalbuminuria, is believed to reflect endothelial dysfunction even in otherwise healthy persons [10-12]. Proteinuria thus might be useful as a indirect marker of systemic capillary leakage. Moreover, it also has been associated with illness severity and mortality on the intensive care unit, thus having a prognostic potential [13,14].

Based on these findings, we hypothesize that low levels of BNP in combination with increased levels of proteinuria reflect inadequate resuscitation due to increased capillary leakage and predict worse outcome in patients with severe burns.

## Methods and methods

### Patients

Between January 2006 and October 2009, all consecutive patients admitted to the burn centre of the Martini Hospital were included. Inclusion criteria were a total burned body surface area (TBSA) ≥ 20% or TBSA between 15 and 20% and inhalation injury. Exclusion criteria were age < 18 years and a life expectancy less than 24 hours.

In total 38 patients were included. Main characteristics at admission are presented in Table 1.

**Table 1:** Characteristics at admission

	Patients (*n *= 38)	Patients with low BNP (*n *= 19)	Patients with high BNP (*n *= 19)
**Age, years**	45 (37 to 60)	46 (31 to 59)	47 (37 to 63)
**Male, n (%)**	28 (74%)	15 (79%)	13 (68%)
**Weight, kg**	87 (74 to 100)	90 (77 to 112)	85 (70 to 99)
**Medical history, n (%)**			
**No CVD, DM or hypertension**	29 (76%)	14 (74%)	15 (79%)
**CVD, DM and/or hypertension**	9 (24%)	5 (26%)	4 (21%)
**Patients with CVD**	5 (13%)	3 (16%)	2 (11%)
**TBSA > 20%, n (%)**	33 (87%)	17 (89%)	16 (84%)
	15 with inhalation	9 with inhalation	6 with inhalation
**TBSA 15 to 20% and inhalation, n (%)**	5 (13%)	2 (11%)	3 (16%)
**Intubation, n (%)**	29 (76%)	14 (74%)	15 (79%)
**TBSA, %**	32 (24 to 42)	35 (24 to 41)	28 (20 to 43)
**Full thickness burn, %**	15 (8 to 25)	17 (8 to 32)	13 (5 to 23)
**Creatinin clearance, ml/min**	139 (88 to 190)	149 (122 to 193)	109 (81 to 192)

Data are presented by median (interquartile range).

CVD, cardiovascular disease; DM, diabetes mellitus; TBSA, total burned body surface area.

All clinical data were collected prospectively on a daily basis. The local research ethics committees gave approval for the study. Informed consent was not deemed necessary.

### Data collection

Retrospective analyses of a prospectively collected database were performed. Data were collected from the day of admission until Day 20 post-burn. At the day of admission the following variables were recorded: gender, age, weight, TBSA, percentage body surface area of full thickness burn, presence of inhalation injury and intubation, trauma mechanism, comorbidity and use of RAAS-inhibitors. Furthermore, at admission and every day at 6.00 am. vital signs (blood pressure and heart rate) were measured. Blood was drawn for several measurements, including BNP which was measured with a two-step chemiluminescent microparticle immunoassay (CMIA) in human EDTA plasma on the ARCHITECT 2000i System (ABBOTT Diagnostic Division; decision threshold for heart failure 100 ng/l); detection limit ≤ 10 ng/l; measurement range 10 to 5,000 ng/l; Total analytical CV at 92.2 ng/l is 4.4%, at 504.3 is 2.7%. As BNP levels increased in the first days of admission and reached a plateau at Day 3 post-burn, patients were divided based on median of BNP level at Day 3.

Urine output was recorded and collected to analyse proteinuria, which was measured with a Microprotein Assay (pyrogallol red and molybdate method) on de SYNCHRON LX20 (Beckman Coulter); reference range < 0.14 g/l; detection limit is 0.06 g/l; measurement range 0.06 to 1.50 g/l; Total analytical CV at 0.17 g/l is 3.8% and at 0.67 g/l is 3.7%. Because of high variability, proteinuria on Days 1 and 2 was averaged.

Every day, dosage and type of catecholamines, the fraction of oxygen in inspired gas and the amount of fluids ordered and actually given were noted. The amount of fluids in the first 48 hours was determined based on the following formulas: from 0 to 8 hours 1.5 ml × weight (kg) × TBSA (%) + 800 ml; from 8 to 24 hours 1 ml × weight (kg) × TBSA (%) + 1,600; from 24 to 48 hours, 1 ml × weight (kg) × TBSA (%) + 3,600 ml. After the first 48 hours, the amount of fluid is corrected based on a minimum urine production of 0.5 ml × weight (kg)/hour. The ordered volumes and the actually received volumes were recorded.

The mean outcome of survival was assessed by the Sequential Organ Failure Assessment (SOFA) score [15]. However, a good clinical condition of a patient has lead to a decrease in frequency of measurements necessary to determine SOFA score. In patients with good clinical condition, interpolated values based on the most recent and subsequent recorded values were used to calculate SOFA-scores.

### Statistical methods

In this small group of patients all variables were not-normally distributed (Shapiro-Wilk test *P-*values < 0.05), except for age. We decided to report all data as median and interquartile range.

Comparisons between the different groups of patients were made by Mann-Whitney tests for continuous variables and by chi-square analysis for categorical variables.

Patients were divided based on median of BNP at Day 3 post-burn (118 ng/l) and median of the averaged proteinuria at Days 1 and 2 (0.9 g/24 hr). The univariate correlation between BNP at Day 3 or averaged proteinuria at Days 1 and 2 and other categorical variables was assessed by Pearson correlation coefficient. Multiple-linear regression analyses were undertaken to determine contributions to BNP levels at Day 3. Potential predictor variables included gender, age, renal function, cardiac disease, total intravenous fluid therapy of day of injury until Day 2, inhalation injury and TBSA. A backwards elimination procedure was used to discard predictor variables with *P *< 0.1 in multiple regression models one by one until a final 'best' model was achieved.

Analyses were performed with SPSS, version 15.0 for Windows (SPSS Inc., Chicago, IL, USA). In addition, we conducted Latent Growth Modeling with MPlus version 6.1 to analyse the influence of time between groups [16]. An alpha of 5% was adopted. Bonferroni-Holm corrections were used for multiple testing of hypotheses.

## Results

### B-type natriuretic peptide

**Figure 1:**
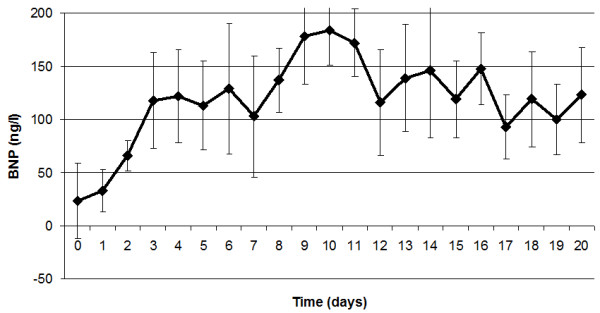
**B-type Natriuretic Peptide (BNP) levels during follow up**. This figure demonstrates the BNP levels during follow-up. A plateau level is reached at Day 3.

Median BNP was 24 (8 to 88) ng/l on day of admission and 33 (18 to 72) ng/l on Day 1 post-burn. BNP increased significantly during the following days. At Day 3 post-burn, a plateau level of BNP was reached (Figure 1).

Based on median BNP level at Day 3 post-burn, patients were divided in those with low BNP and those with high BNP levels. Baseline characteristics did not differ between the two groups (Table 1). For patients with high BNP levels, less fluid volumes were ordered at days 3, 4 and 5 (*P *< 0.05). Received fluid volumes also were less from Day 3 to Day 10 (*P *< 0.05).

**Figure 2:**
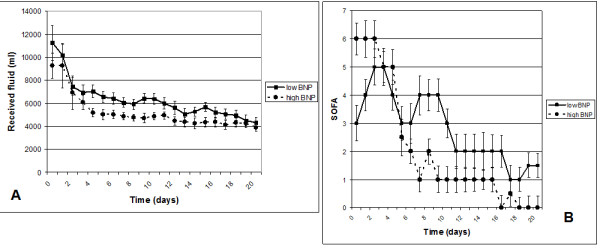
**Total received fluid and SOFA scores in patients with high and low BNP**. **A**. Received fluid, **B**. Sequential Organ Failure Assessment (SOFA) scores. Dashed line presents patients with a high B-type Natriuretic Peptide (BNP), closed line patients with a low BNP.

Levels of BNP at Day 3 post-burn were negatively correlated to the amounts of received fluids at days 4, 5, 6, 17 and 20 (r = -0.46, -0.39, -0.49, -0.39 and -0.47 respectively, all with *P *< 0.05 with Bonferroni-Holm correction). Levels of BNP were negatively correlated to SOFA scores at days 16, 18 and 20 (r = -0.45, -0.54 and -0.42 respectively, all with *P *< 0.05 with Bonferroni-Holm correction). Furthermore, patients with a high BNP levels had less morbidity, reflected by lower SOFA scores (Figure 2).

In univariate analysis, no correlations were found between levels of BNP with age (r = 0.28, *P *= 0.13), TBSA (r = -0.25, *P *= 0.17), weight (r = -0.20, *P *= 0.27), creatinin clearance (r = -0.23, *P *= 0.27), urine production (r = 0.27, *P *= 0.14) or heart rate (r = -0.20, *P *= 0.27). Furthermore, levels of BNP did not differ between gender (female vs male, 199 (101 to 402) ng/l vs 114 (36 to 200) ng/l, *P *= 0.35). BNP levels tended to be lower in patients with inhalation injury (106 (30 to 178) ng/l, *n *= 18 versus 225 (104 to 385) ng/l, *n *= 20, *P *= 0.053).

Multiple-linear regression analyses were performed to determine contributions to BNP levels at Day 3. Potential predictor variables included gender, age, renal function, cardiac disease, total intravenous fluid therapy of day of injury until Day 2, inhalation injury and TBSA. Backward elimination procedure revealed that age, inhalation injury and total fluid therapy until Day 2 were the most important determinants of BNP levels at Day 3 (R square = 0.81, beta = 0.44, *P *= 0.02, beta = -0.510, *P *= 0.02, and beta = -0.37, *P *= 0.07, respectively).

Latent Growth Modelling showed no difference in initial SOFA scores for the groups with high or low BNP at Day 3 (predictive posterior *P*-value = .36). The high BNP group is reaching better SOFA scores earlier (regression weight for slope difference is -0.22 per day, *P *= .03, one-tailed).

### Proteinuria

Because of expected high variability, proteinuria on Days 1 and 2 was averaged (median 0.91 (0.59 to 2.75) g/24 hr). Averaged proteinuria on Days 1 and 2 was positively correlated to SOFA score at Days 0, 1 and 2 (r = 0.35, 0.47 and 0.45 respectively, all with *P *< 0.05 with Bonferroni-Holm corrections). No correlations were found between levels of averaged proteinuria with age, TBSA, weight and creatinin clearance. Proteinuria tended to be higher in patients with inhalation injury (1.74 (0.63 to 4.96) g/24 hr, *n *= 20, versus 0.64 (0.5 to 1.07) g/24 hr, *n *= 18, *P *= 0.08). Latent Growth Modeling showed differences in initial SOFA scores for the groups with high or low proteinuria. The SOFA score of the high proteinuria group was on average 2.2 points higher (posterior predictive *P*-value = < .0001). The effect of time was in both groups equal (*P *= .225)

### Combining BNP and proteinuria

**Figure 3:**
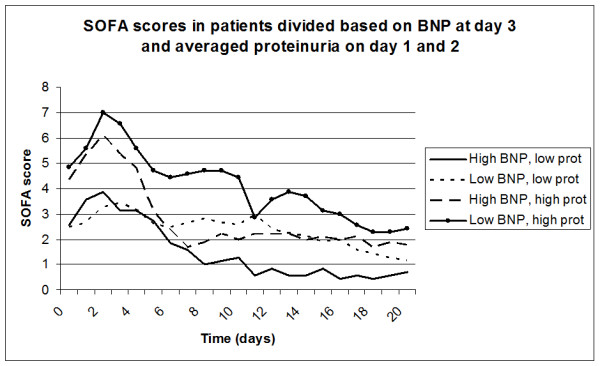
**SOFA scores in groups of patients divided based on median BNP and median proteinuria**.

By dividing all patients based on the median of BNP level at Day 3 and the median of averaged proteinuria at Days 1 and 2, the four different groups did not differ in age, TBSA, inhalation trauma, use of RAAS-inhibitors or number of deceased patients (Table 2). Patients with high BNP and low proteinuria had significantly lower SOFA scores during whole follow-up compared to those patients with low BNP and high proteinuria. Intermediate SOFA scores were seen in the two other groups (Figure 3).

**Table 2:** Characteristics of the different groups divided based on median BNP and median proteinuria

	High BNP, low prot (*n *= 7)	Low BNP, low prot (*n *= 12)	High BNP, high prot (*n *= 12)	Low BNP, high prot (*n *= 7)
**Age, years**	43 (35 to 63)	55 (31 to 63)	48 (39 to 61)	38 (37 to 46)
**Male, n (%)**	5 (71%)	8 (75%)	8 (75%)	7 (100%)
**Weight, kg**	70 (70 to 100)	91 (78 to 117)	87 (76 to 97)	90 (76 to 100)
**TBSA > 20%, n (%)**	6 (86%)	10 (83%)	10 (83%)	7 (100%)
**Inhalation, n (%)**	2 (29%)	6 (50%)	7 (58%)	6 (86%)
**TBSA, %**	28 (25 to 45)	31 (21 to 40)	32 (20 to 43)	37 (30 to 45)
**Creatinin clearance, ml/min**	95 (80 to 148)	148 (116 to 188)	122 (81 to 200)	156 (131 to 206)
**Dead, n (%)**	0	1 (8%)	3 (25%)	0
**SOFA at Day 20**	0 (0 to 1)	0 (0 to 3)	1 (0 to 3.5)	3 (1 to 4)

Data are presented by median (interquartile range)

(Inhalation: *P *likelihood *P *= 0.157, lineair *P *= 0.03)

SOFA, sequential organ failure assessment; TBSA, total burned body surface area

## Discussion

This is the first study which combines levels of BNP and proteinuria in patients with severe burns to investigate whether these clinical parameters could be used as markers of resuscitation and prognosis.

Rapid and aggressive fluid resuscitation is crucial in patients with severe burn injury. However, no formula is available yet which determines the exact burn victim's fluid requirements. Nowadays, the well known formulas as the Parkland formula or the modified Brooke formula are used. However, these only guide the initiation of fluid resuscitation (for example, the first 24 hours) and especially the amounts of fluid as calculated by the Parkland formula is subject to discussion [17,18]. The requirement of fluids of each individual patient, especially after the first 48 hours, is difficult to determine with these formulas. Therefore, adjustments to estimated fluid requirements must be made based upon a patient's physiologic response to resuscitation. Most often urine production is used. Other clinical signs of volume status, such as heart rate, blood pressure, capillary refill, and colour of uninjured skin are also taken into account. Specific laboratory measurements for adequacy of resuscitation are mixed venous blood gas and lactate. Invasive monitoring such as central venous pressure may also be useful for monitoring fluid resuscitation, but is invasive, has an increased risk of infection and is not always available. Despite all these different markers, it still remains very difficult to determine the optimal amount of fluid in each individual patient in clinical practice. BNP is an interesting marker of fluid resuscitation as it is secreted from the myocardium under increased wall stretch. BNP is nowadays used in the diagnosis of heart failure [5-8]. We investigated whether BNP levels can be used to monitor fluid balance in patients with severe burns. We hypothesize that in case of resuscitation and no loss of fluid into the third space, BNP levels increase. Indeed, in a few studies with a small number of patients increasing levels of BNP have been demonstrated during resuscitation [3,4]. In our study BNP levels increased during the follow-up, especially during the first three days of resuscitation in which the highest amounts of fluid were administered. Those patients who received a smaller amount of fluids, and thus were hemodynamically stable since the amount of fluids was determined based on adequate urine production and blood pressure, had increased BNP levels. Probably, these higher levels of BNP reflect fluid distribution to be more present in the intravascular compartment due to less capillary leakage. Furthermore, the finding that BNP levels were lower in patients with inhalation injury, who frequently require larger than predicted fluid resuscitation volumes, supports the hypothesis that low BNP levels may be due to more severe injury and increased fluid needs, which are not being met during resuscitation. To translate this into clinical practice, this might support higher resuscitation volumes in burn patients with inhalation injury compared to patients without inhalation injury.

Several factors are known to correlate with BNP, such as age, gender, renal function and cardiac history. In this study, BNP levels were not influenced by renal function, since in most patients renal function remained normal during follow-up and did not correlate with BNP (creatinine clearance Day 3 vs BNP level Day 3, r = -0.23, *P *= 0.27). Furthermore, only five patients had a cardiac history. These patients were equally divided between the groups and did not influence BNP levels. No correlation was found between gender and levels of BNP, in contrast to what is described in the literature [19]. A possible explanation, besides the rather small patient group, is the fact that hyperdynamic circulation and changed fluid status influence levels of BNP more than renal function, history of cardiac disease or gender in patients with severe burns. Increased BNP levels have been described in septic patients, which is caused by several factors; for example, inflammation, myocardial dysfunction, severity of global tissue hypoxia, fluid management, vasoactive drugs and renal dysfunction [7,20]. Of course, it is interesting to evaluate whether our patients with high BNP levels had an increased cardiac output state compared to those with low BNP levels. As no invasive measurements were available, heart rate was used as a marker of a hyperdynamic state. No correlation was found between levels of BNP at Day 3 with heart rate at Day 3 (r = -0.20, *P *= 0.27), indicating that other variables, including fluid status, might influence levels of BNP even more.

BNP has been shown to be prognostically negative in the intensive care population and has been called the death hormone [21]. However, predicting mortality in the intensive care unit has been inconsistent, with several studies showing that BNP levels correlate with mortality and others that do not [20-23]. Many conditions and therapies common in intensive care patients can affect BNP levels. Also, there is a high variability in the characteristics of intensive care patients enrolled in these studies. It might be hypothesized that in burn patients the worst outcome is correlated with extensive capillary leakage, which is reflected by low BNP levels despite a high amount of fluid resuscitation.

Proteinuria is an interesting marker of survival in critical ill patients [13,14]. In this study, patients with higher average proteinuria at Days 1 and 2 have higher SOFA scores reflecting a worse outcome. Moreover, a trend was found between proteinuria and inhalation injury. In the literature, there are conflicting data on the association between outcome and microalbuminuria in patients with severe burns. Yew *et al. *[24] concluded that microalbuminuria is a strong predictor of mortality; a more recent study did not find a correlation between microalbuminuria and outcome in burn injury [25]. At this point, it is not known whether proteinuria or microalbuminuria is a better prognostic marker or what the best interpretation would be. We decided to use proteinuria, as Sviridov *et al. *proposed, instead of albuminuria in patients with severe burns, since the composition of urinary proteins is disturbed and unusual after burn injury [26]. In future studies it would be interesting to include proteinuria as well as albuminuria.

Proteinuria and, especially, microalbuminuria have been described as markers of endothelial dysfunction c.q. systemic capillary leakage [10,11,27,28]. As capillary leakage influences the individual need of resuscitation, it would be very interesting to combine BNP and proteinuria. It could be hypothesized that patients with high proteinuria have more capillary leakage, and thus no increase in BNP levels. Indeed, this study showed that patients with low BNP and high proteinuria performed worse than patients with high BNP and low proteinuria.

This study has some limitations. A rather small number of patients was included. Furthermore, there were missing data; for example, when patients had good clinical conditions fewer laboratory measurements were performed, resulting in difficulties in calculating SOFA scores. Interpolated values were used based on the most recent and subsequently recorded values to prevent bias. Otherwise calculation of SOFA-scores would only have been possible for patients who were in poor condition.

Concerning the measurement of proteinuria, many variables, which were not included, are known to influence proteinuria, (for example, fever and use of aminoglycosides). It would be interesting to include these variables in future investigations. No laboratory measurements, such as of lactate or invasive measurements, were determined to confirm that BNP levels reflect the hemodynamic situation of the patient. In larger cardiac studies, BNP generally correlates with cardiac function [29]. Furthermore, levels of BNP have been demonstrated to correlate to mean pulmonary capillary wedge pressure as an indicator of left ventricular end-diastolic pressure in patients with acute dyspnoea [8]. However, in a more general intensive care population the correlation between BNP and invasive measurements is rather poor [30]. Also, Papp *et al. *did measure BNP and invasive measurements in nine burn patients, and no associations were found [4]. On the other hand, the association of a high BNP and the lower received fluid volume found in this study suggests that BNP can be used as a tool along with other clinical markers, including urine production and blood pressure, in the individual patient to adjust fluid orders to actual demands. Further research should be performed to confirm this hypothesis.

## Conclusions

Nowadays, in clinical practice the amount of resuscitation fluids in burn patients are calculated by formulas such as the Parkland formula, which is based on weight and TBSA. After the first 24 hours (or 48 hours) the amount of fluid is adjusted most often based on clinical parameters, such as urine production, blood pressure and heart rate. We propose that a combination of BNP levels and proteinuria might be used to individualize the administration of the amounts of fluid and to predict outcome in patients with severe burns.

## Key messages

• It is difficult to adjust fluid balance adequately in patients with severe burns due to various physical changes.

• B-type Natriuretic Peptide (BNP) is emerging as a potential marker of hydration state and might be used to individualize the administration of the amounts of fluid in patients with severe burns.

• Proteinuria might reflect systemic capillary leakage and is also associated with illness severity and mortality on the intensive care unit.

• In combination with low proteinuria, possibly reflecting minimal capillary leakage, a high BNP level was associated with a better outcome.

• Thus, BNP and proteinuria might have prognostic potential in severely burned patients.

## Abbreviations

BNP: B type natriuretic peptide; CMIA: chemiluminescent microparticle immunoassay; CVD: cardiovascular disease; DM: diabetes mellitus; SOFA: sequential organ failure assessment; TBSA: total burned body surface area.

## Competing interests

The authors declare that they have no competing interests.

## Authors' contributions

KdL performed the statistical analysis and drafted the manuscript. MN participated in the data collection and helped to draft the manuscript. AN assisted in the statistical analysis and JE collected the data. GB participated in the design of the study and helped to draft the manuscript. WJ conceived of the study and participated in its design, and helped to draft the manuscript. All authors read and approved the final manuscript.

## References

[B1] Huang QB Zhao M Chen B Li Q The regulation of vascular permeability in severe shock Mol Mech Severe Shock 2009 157 173

[B2] Demling RH The burn edema process: current concepts J Burn Care Rehabil 2005 26 207 227 15879742

[B3] Friese RS Dineen S Jennings A Pruitt J McBride D Shafi S Frankel H Gentilello LM Serum B-type natriuretic peptide: a marker of fluid resuscitation after injury? J Trauma 2007 62 1346 1350 10.1097/TA.0b013e31804798c3 17563646

[B4] Papp A Uusaro A Parviainen I Hartikainen J Ruokonen E Myocardial function and haemodynamics in extensive burn trauma: evaluation by clinical signs, invasive monitoring, echocardiography and cytokine concentrations. A prospective clinical study Acta Anaesthesiol Scand 2003 47 1257 1263 10.1046/j.1399-6576.2003.00235.x 14616324

[B5] Lansink-Hartgring AO Eshuis J Nieuwenhuis MK Beerthuizen GI Janssen WM Adult respiratory distress syndrome or congestive heart failure in severe burn: A role for brain natriuretic peptide Burns 2009 36 e87 90 20036070 10.1016/j.burns.2009.10.010

[B6] Omland T Advances in congestive heart failure management in the intensive care unit: B-type natriuretic peptides in evaluation of acute heart failure Crit Care Med 2008 36 S17 S27 10.1097/01.CCM.0000296266.74913.85 18158473

[B7] Tsai SH Lin YY Chu SJ Hsu CW Cheng SM Interpretation and use of natriuretic peptides in non-congestive heart failure settings Yonsei Med J 2010 51 151 63 10.3349/ymj.2010.51.2.151 20191004 PMC2824858

[B8] Zhao SQ Hu YM Li Q Liu XR Wang M Zhang WY Wu T Nie XL Zhao N Wang LR The clinical value of rapid assay for plasma B-type natriuretic peptide in differentiating congestive heart failure from pulmonary causes of dyspnoea Int J Clin Pract 2008 62 214 220 18081799 10.1111/j.1742-1241.2007.01591.x

[B9] Vlachou E Gosling P Moiemen NS Microalbuminuria: a marker of systemic endothelial dysfunction during burn excision Burns 2008 34 241 246 10.1016/j.burns.2007.03.021 17698293

[B10] Deckert T Feldt-Rasmussen B Borch-Johnsen K Jensen T Kofoed-Enevoldsen A Albuminuria reflects widespread vascular damage. The Steno hypothesis Diabetologia 1989 32 219 226 10.1007/BF00285287 2668076

[B11] Stehouwer CD Henry RM Dekker JM Nijpels G Heine RJ Bouter LM Microalbuminuria is associated with impaired brachial artery, flow-mediated vasodilation in elderly individuals without and with diabetes: further evidence for a link between microalbuminuria and endothelial dysfunction--the Hoorn Study Kidney Int Suppl 2004 92 S42 S44 15485416 10.1111/j.1523-1755.2004.09211.x

[B12] Jensen JS Feldt-Rasmussen B Strandgaard S Schroll M Borch-Johnsen K Arterial hypertension, microalbuminuria, and risk of ischemic heart disease Hypertension 2000 35 898 903 10775558 10.1161/01.hyp.35.4.898

[B13] Gopal S Carr B Nelson P Does microalbuminuria predict illness severity in critically ill patients on the intensive care unit? A systematic review Crit Care Med 2006 34 1805 1810 10.1097/01.CCM.0000217922.75068.EA 16625124

[B14] Gosling P Czyz J Nightingale P Manji M Microalbuminuria in the intensive care unit: Clinical correlates and association with outcomes in 431 patients Crit Care Med 2006 34 2158 2166 10.1097/01.CCM.0000228914.73550.BD 16775565

[B15] Vincent JL Moreno R Takala J Willatts S De Mendonça A Bruining H Reinhart CK Suter PM Thijs LG The SOFA Sepsis-related Organ Failure Assessment. score to describe organ dysfunction/failure. On behalf of the Working Group on Sepsis-Related Problems of the European Society of Intensive Care Medicine Intensive Care Med 1996 22 707 710 10.1007/BF01709751 8844239

[B16] Muthén LK Muthén BO Mplus User's Guide 1998 6 Los Angeles: Muthen & Muthen

[B17] Bak Z Sjoberg F Eriksson O Steinvall I Janerot-Sjoberg B Hemodynamic changes during resuscitation after burns using the Parkland formula J Trauma 2009 66 329 336 10.1097/TA.0b013e318165c822 19204504

[B18] Blumetti J Hunt JL Arnoldo BD Parks JK Purdue GF The Parkland formula under fire: is the criticism justified? J Burn Care Res 2008 29 180 186 10.1097/BCR.0b013e31818cf8b8 18182919

[B19] McLean AS Huang SJ Nalos M Tang B Stewart DE The confounding effects of age, gender, serum creatinine, and electrolyte concentrations on plasma B-type natriuretic peptide concentrations in critically ill patients Crit Care Med 2003 31 2611 2618 10.1097/01.CCM.0000094225.18237.20 14605532

[B20] Sturgess DJ Marwick TH Joyce C Jenkins C Jones M Masci P Stewart D Venkatesh B Prediction of hospital outcome in septic shock: a prospective comparison of tissue Doppler and cardiac biomarkers Crit Care 2010 14 R44 10.1186/cc8931 20331902 PMC2887156

[B21] Kotanidou A Karsaliakos P Tzanela M Mavrou I Kopterides P Papadomichelakis E Theodorakopoulou M Botoula E Tsangaris I Lignos M Ikonomidis I Ilias I Armaganidis A Orfanos SE Dimopoulou I Prognostic importance of increased plasma amino-terminal pro-brain natriuretic peptide levels in a large noncardiac, general intensive care unit population Shock 2009 31 342 347 10.1097/SHK.0b013e31818635b6 18791494

[B22] Ginsberg F Topalian S Natriuretic peptides: biomarkers not predictive in the intensive care unit Crit Care Med 2007 35 1194 1195 10.1097/01.CCM.0000260056.54558.29 17413786

[B23] McLean AS Huang SJ Hyams S Poh G Nalos M Pandit R Balik M Tang B Seppelt I Prognostic values of B-type natriuretic peptide in severe sepsis and septic shock Crit Care Med 2007 35 1019 1026 10.1097/01.CCM.0000259469.24364.31 17334249

[B24] Yew WS Pal SK Correlation of microalbuminuria and outcome in patients with extensive burns Br J Anaesth 2006 97 499 502 10.1093/bja/ael211 16885170

[B25] Cochran A Dong L Edelman LS Roberts WL Ballard J Privette A Morris SE Saffle JR Microalbuminuria in acute burn injury J Burn Care Res 2008 29 176 179 10.1097/BCR.0b013e31818cf8aa 18182918

[B26] Sviridov D Owen WE Roberts WL Edelman LS Drake SK Hortin GL Proteinuria without albuminuria: urinary protein excretion by a subset of patients with burn injuries Clin Chim Acta 2009 403 42 46 10.1016/j.cca.2009.01.012 19361474 PMC5606198

[B27] Jensen JS Borch-Johnsen K Jensen G Feldt-Rasmussen B Microalbuminuria reflects a generalized transvascular albumin leakiness in clinically healthy subjects Clin Sci (Lond) 1995 88 629 633 7634745 10.1042/cs0880629

[B28] Paisley KE Beaman M Tooke JE Mohamed-Ali V Lowe GD Shore AC Endothelial dysfunction and inflammation in asymptomatic proteinuria Kidney Int 2003 63 624 633 10.1046/j.1523-1755.2003.00768.x 12631127

[B29] Richards M Nicholls MG Espiner EA Lainchbury JG Troughton RW Elliott J Frampton CM Crozier IG Yandle TG Doughty R MacMahon S Sharpe N Comparison of B-type natriuretic peptides for assessment of cardiac function and prognosis in stable ischemic heart disease J Am Coll Cardiol 2006 47 52 60 10.1016/j.jacc.2005.06.085 16386664

[B30] Forfia PR Watkins SP Rame JE Stewart KJ Shapiro EP Relationship between B-type natriuretic peptides and pulmonary capillary wedge pressure in the intensive care unit J Am Coll Cardiol 2005 45 1667 1671 10.1016/j.jacc.2005.01.046 15893185

